
JOURNAL INFORMATION
==============================
NLM Title Abbreviation: ScientificWorldJournal
Journal ID: ScientificWorldJournal
NLM Journal ID: 101131163
Journal ID: TSWJ

The Scientific World Journal

ISSN: 2356-6140
EISSN: 1537-744X
Publisher: Wiley

ARTICLE INFORMATION
==============================
PMCID: PMC6009442
PMID: 29973835
DOI: 10.1100/tsw.2002.60
Article ID: 709383
Article version: 1
Subjects: Short Report

Engineering Polyketide Synthases

Katz Leonard
Kosan Biosciences, Inc., USA
Publication date: 2002
Electronic publication date: 2002 Jan 16
Volume: 2
First page: 127
Last page: 129
Copyright: Copyright © 2002 Leonard Katz.
Copyright year: 2002
License: This is an open access article distributed under the Creative Commons Attribution License, which permits unrestricted use, distribution, and reproduction in any medium, provided the original work is properly cited.
License URL: https://creativecommons.org/licenses/by/3.0/

==============================
Advances in Gene Technology: The Genome and Beyond –
Structural Biology for Medicine (Proceedings of the 2002 Miami
*Nature Biotechnology* Winter Symposium)
*TheScientificWorld* 2002, 2(S2), 127–129
ISSN 1532-2246; DOI 10.1100/tsw.2002.60

### ENGINEERING POLYKETIDE SYNTHASES

Leonard Katz
Kosan Biosciences, Inc.

Complex polyketides, exemplified by the molecules shown in Fig. 1, are produced mainly from a group of mycelia-forming bacteria belonging to the actinomycete family and have applications in medicine or agriculture. Worldwide sales of polyketides exceed $15 B annually. Up to now, all marketed polyketide drugs are either the natural product or a chemically modified version thereof. The need for novel antibiotics, anticancers, and antifungals has underscored great interest in these compounds and the search for new ways to generate chemical diversity around these natural products.

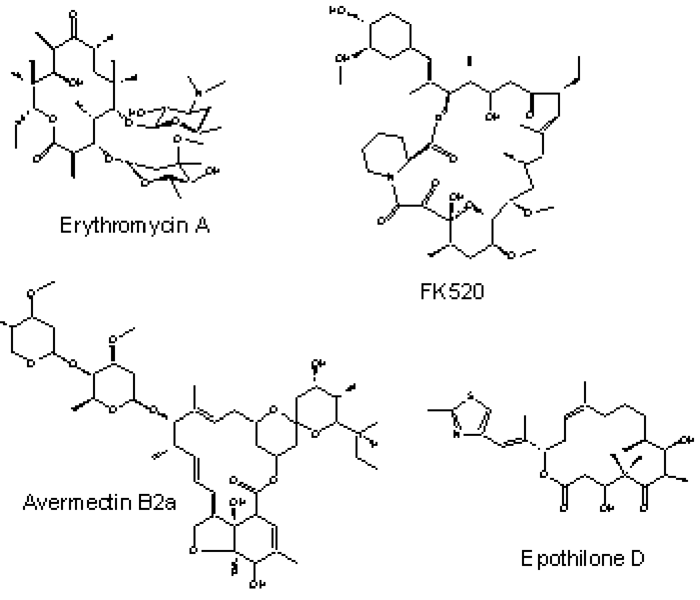

Fig. 1 Structures of complex polyketides

Though diverse in structure and pharmacological properties, these compounds are synthesized by processes that are all remarkably similar. Erythromycin, the prototypical complex polyketide is composed of a 14-membered macrolactone ring to which are attached two deoxysugars. The backbone is made through the successive condensation of a propionyl CoA (pCoA) starter unit and six methylmalonyl CoA units as extenders in a manner that resembles fatty acid biosynthesis. Unlike typical fatty acid synthesis, in polyketide biosynthesis, the starter can vary from acetyl CoA: e.g., pCoA in erythromycin, isobutyryl CoA in avermectin, dihydroxycyclohexanyl CoA in FK520, etc., and the extenders can vary from malonyl CoA (mCoA): e.g., mmCoAs in erythromycin, and mCoA , mmCoA, ethylmalonyl CoA, and methoxymalonyl CoA in FK520.

Since only two carbons are added to the nascent acyl backbone, the additional carbons in methylmalonate, ethylmalonate, or methoxymalonate form the methyl or ethyl or methoxy side chains seen in erythromycin, FK520, and other complex polyketides. In further contrast to fatty acid synthesis, wherein the β-ketone formed in the nascent acyl chain at each condensation step is fully reduced to the resulting methylene group at nearly every step in chain elongation, reductions at specific points in chain growth of polyketides often do not take place at all or are not fully completed, resulting in the generation of the keto or hydroxyl groups or double bonds in the completed molecules as depicted in the structures shown in Fig. 2. Thus the chemical diversity seen across the family of some 6000 complex polyketides comes from (1) the starter utilized, (2) the extender employed, (3) the fate of the β-carbonyl generated at each step of chain growth, (4) the number of condensation steps (erythromycin has 6; FK520 has 11), (5) stereochemistry of the side chains, and (6) postpolyketide modifications including glycosylation, methylation, hydroxylation, etc.

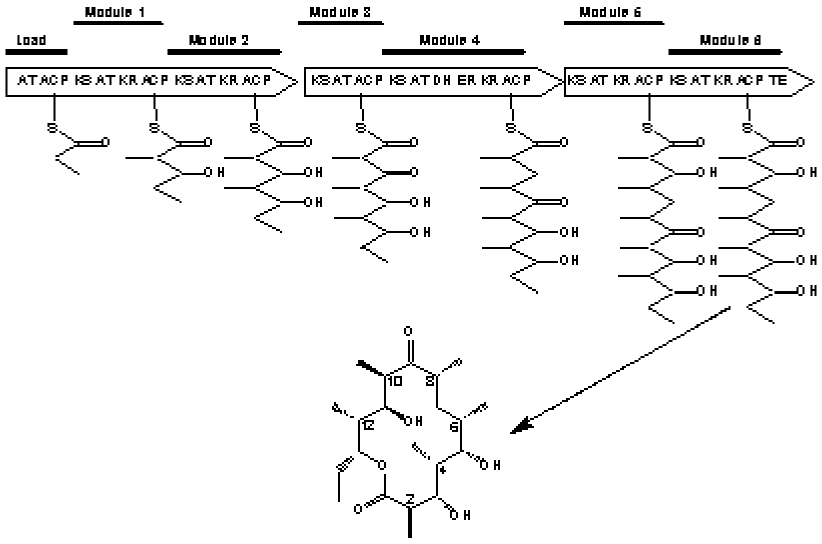

Fig. 2. The erythromycin polyketide synthase and growth of the polyketide chain

The polyketide backbone is produced by the enzyme complex known as the polyketide synthase (PKS). As shown in Fig. 2 for the production of 6-deoxyerythronolide B (6-dEB), the polyketide backbone of erythromycin, each step of the synthesis is determined by a module, which is itself composed of enzymatic domains that each carry out one reaction in chain growth: KS – condensation; KR – β-ketoreduction; DH – dehydration; ER – enoylreduction. The ACP domains carry the growing chain in a thioester linkage to the PKS and present it to the reductive domains for β-carbonyl processing. The AT domain determines the extender unit (or starter for the loading module) utilized. For erythromycin (ery) there are 6 modules for 6 condensations to produce a 14-membered macrolide. Similarly, for FK520, which requires 11 condensations, the PKS is composed of 11 modules; the epothilone PKS has 9 modules, etc., although the epothilone and FK520 PKSs are linked to nonribosomal peptide synthetases acting either during (epothilone) or after (FK520) acyl chain growth. Thus, the structure of the polyketide is programmed at the level of the functional domains in the PKS, which itself is determined by the sequence of the PKS genes.

It is possible to introduce specific changes into the PKS genes to change the structure of the polyketide at the corresponding position in the ring. For example, changes introduced at module 6 of the *ery* PKS gene *eryAIII* affect the structure at carbons 2 or 3, changes in module 4 affect the structure at C6 or C7, etc. Thus, hydroxyl groups at C5 and C3 have been replaced by keto groups in 6-dEB by the introduction of function disabling mutations in the KR domains of modules 5 and 6, respectively. Similarly, the AT domains of several of the erythromycin modules have been exchanged for those from other PKS genes that specify the incorporation of m-CoA in place of the usual mm-CoA, to result in the production of the corresponding desmethyl 6-dEB derivatives: some have been further converted to erythromycin analogs. Changes have also been introduced in other PKS genes to produce similar types of polyketide analogs: e.g. desmethoxyFK520 compounds.

The cloning of the *ery* PKS genes from the chromosome of the producing bacterium, *Saccharopolyspora erythraea* into plasmids capable of replicating in both *E. coli* and *Streptomyces coelicolor* has enabled a combinatorial approach to creating erythromycin analogs carrying multiple chemical changes. Each of the three *ery* PKS genes has been cloned on separate plasmids wherein different mutations or exchanges were introduced. By combining different variants of each gene in *S. coelicolor*, greater than 50 6-dEB analogs containing 1, 2, or 3 alterations in their structure have been produced. Many of the changes produced are difficult or impossible to achieve by direct chemical modification. Further changes have been made by combining genetically altered polyketide backbones with deoxysugars or by adding or removing postpolyketide modification enzymes: hydroxylases or methylases. Finally, in the case of 6-dEB, the natural diketide formed after the first condensation has been replaced by the addition of novel diketides as thioesters to an *ery* PKS point mutant blocked only in the first condensation step (KS1 null strain) to produce a series of novel analogs which carry alterations of the side chain at C13.

**REFERENCES**

1. Katz, L. and McDaniel, R. (1999) Med. Res. Rev. 6, 543–558.
2. Khosla, C., Gokhale, R.S., Jacobsen, J.R., and Cane, D.E. (1999) Annu. Rev. Biochem. 68, 219–253.
3. Rodriguez, E. and McDaniel, R. (2001) Curr. Opin. Microbiol. 4, 526–534.
